# Therapeutic effectiveness of conditioned medium derived from adipose tissue mesenchymal stem cells and dehydroepiandrosterone in a rat model of spinal cord injury

**DOI:** 10.1016/j.ibneur.2025.12.010

**Published:** 2025-12-22

**Authors:** Farrokh Modarresi, Gholam Reza Kaka, Mehdi Raei, Fatemeh Rezaei-Tazangi

**Affiliations:** a Baqiyatallah Research Center for Gastroenterology and Liver Diseases (BRCGL, Baqiyatallah University of Medical Sciences, Tehran, Iran; b Faculty of Medicine, Baqiyatallah University of Medical Sciences, Tehran, Iran; c Neuroscience Research Center, Baqiyatallah University of Medical Sciences, Tehran, Iran; d Department of Anatomy, Baqiyatallah University of Medical Sciences, Tehran, Iran; e Department of Epidemiology and Biostatistics, Faculty of Health, Baqiyatallah University of Medical Sciences, Tehran, Iran; f Department of Anatomy, School of Medicine, Fasa University of Medical Sciences, Fasa, Iran

**Keywords:** Spinal cord injury, Conditioned medium from adipose-derived mesenchymal stem cells, Dehydroepiandrosterone

## Abstract

**Background and objective:**

Spinal cord injury (SCI) is a severe neurological disorder that leads to significant complications, including loss of bladder/bowel control and increased infection risk. The current standard treatment involves methylprednisolone administration and surgical decompression, but finding an effective therapy with minimal side effects remains a major challenge. This study aimed to investigate the effects of an optimized conditioned medium derived from rat adipose-derived mesenchymal stem cells (AD-MSCs) and dihydroepiandrosterone (DHEA) on behavioral indices, oxidative stress, stereological parameters, and histopathological outcomes in rats with compressive spinal cord injury (SCI).

**Material and methods:**

In this study, 60 adult female rats were randomly divided into five groups: Sham group (laminectomy + intraperitoneal injection of 1 % dimethyl sulfoxide [DMSO], 200 µL for seven consecutive days), SCI-induced group (SCI induction + intraperitoneal injection of 1 % DMSO, 200 µL for seven consecutive days), Treatment group 1 (SCI induction + intraperitoneal injection of DHEA [30 mg/kg] dissolved in 1 % DMSO for seven consecutive days), Treatment group 2 (SCI induction + intraperitoneal injection of conditioned medium [200 µL] for seven consecutive days), Treatment group 3 (SCI induction + intraperitoneal injection of DHEA [30 mg/kg] dissolved in 1 % DMSO followed by conditioned medium [200 µL] for seven consecutive days). Behavioral assessments were performed using the Basso, Beattie, and Bresnahan (BBB) locomotor rating scale and the rotarod test. Additionally, the levels of antioxidant enzymes—catalase, glutathione (GSH), superoxide dismutase (SOD), and the lipid peroxidation marker malondialdehyde (MDA)—were measured using respective assay kits. For stereological evaluation (to estimate neuronal and non-neuronal cell counts, gray and white matter volumes, spinal cord volume, and lesion area) and histopathological assessment (to evaluate inflammation, necrosis, and hemorrhage indices), tissue samples were stained with Cresyl Violet.

**Results:**

The findings revealed that in the SCI-induced group, motor function, neuronal cell number in spinal gray matter, non-neuronal cell number in the spinal cord, white and gray matter volumes, total spinal cord volume, and levels of catalase, GSH, and SOD were significantly reduced compared to the sham group. Conversely, the spinal lesion volume and MDA levels were elevated. Treatment with DHEA, AD-MSC-conditioned medium, or their combination reversed these effects. Notably, the combined treatment group exhibited more pronounced therapeutic improvements compared to monotherapy groups.

**Conclusion:**

The administration of DHEA and AD-MSC-conditioned medium, particularly in combination, appears to enhance motor function, elevate antioxidant enzyme activity, reduce lipid peroxidation, improve spinal cord structural parameters (volume and cell counts), and ameliorate pathological markers in an animal model of compressive SCI.

## 1 Introduction

Spinal cord injury (SCI) begins with an initial lesion, which is quickly followed by a compilation of molecular and cellular occurrences, leading to a secondary lesion detrimental to functional restoration and nerve repair. The deterioration of the primary lesion involves inflammatory responses, the formation of free radicals, glutamate excitotoxicity, and damage to both neurons and oligodendroglia. Over time, necrosis extends to nearby tissues, resulting in the formation of a cystic cavity. Additionally, the spontaneous axonal regeneration that begins is hindered by an environment rich in glial scar and myelin-associated inhibitors (Quadri et al., 2020). Regarding this matter, various preclinical studies have been accomplished to enhance functional recovery, concentrating on several parameters: management of inflammation, preservation of neural tissue, potentiation of axonal regeneration through regulation of the injured environment, and enhancement of remyelination (Hu et al., 2023).

One of the therapeutic strategies for SCI is the use of steroids like methylprednisolone (MP). MP is the FDA-approved drug for SCI; however, the effectiveness of early high-dose MP therapy in SCI is not well-documented (Bracken et al., 2000). MP administration may elevate the risk of complications after surgical stabilization following damage (Molano et al., 2002), and the overall outcomes reported from MP therapy by a lot of practitioners are not satisfactory yet (Short et al., 2000). Therefore, research emphasizes alternative steroids with reduced side effects. Molecules targeting various signaling pathways related to secondary injury are especially noteworthy. Neurosteroids might belong to this category of molecules. Dehydroepiandrosterone (DHEA), a weak androgen, serves as an inactive precursor of androgen production in the gonads and adrenal glands. It is a potent neuroactive neurosteroid in various central nervous system (CNS) pathophysiology models, modulating several neurotransmitter systems and having neuroprotective properties (Borowicz et al., 2011). Studies have demonstrated that DHEA treatment improves functional recovery after SCI (Fiore et al., 2004).

Another developed therapeutic strategy for SCI is cell transplantation. Mesenchymal stem cells (MSCs) are among the most promising strategies due to their easy isolation, preservation, and beneficial properties. However, they continue to encounter specific limitations, such as a decrease in differentiation potential, the triggering of host immune responses, and challenges in delivery. The toxic environment of the acute SCI lesion also poses difficulties for cellular engraftment (Bahmanpour et al., 2019, Donnelly et al., 2012, Cofano et al., 2019). It was shown that the genuine therapeutic effects of MSCs depend on their released secretomes, which contain a soluble component made up of proteins, growth factors, cytokines, as well as a vesicular component consisting of microvesicles and exosomes (Rezaei-Tazangi et al., 2020, Marote et al., 2016). In light of this information, it appears that the secretomes produced by MSCs may provide greater advantages for tissue repair and regeneration than the stem cells themselves. So, recent studies focus on conditioned medium derived from stem cells for SCI treatment (Arefnezhad et al., 2024).

It was shown that adipose tissue MSCs (AD-MSCs) had stronger proliferation and culture viability under hypoxic conditions than bone marrow MSCs (BM-MSCs) and better tolerance to H_2_O_2_-induced oxidative stress and serum-free medium stress. Likewise, when transplanted into regions of spinal cord injury *in vivo*, AD-MSCs exhibited a greater survival rate post-transplantation. Thus, AD-MSCs are a promising alternative to BM-MSCs for treating severe spinal cord injury (Takahashi et al., 2018).

According to the evidence presented, we chose the AD-MSCs' conditioned medium (secretome) to improve SCI *in vivo*. As far as we know, no prior research has studied the impact of AD-MSC conditioned medium in SCI. Due to the intricate relationships among various molecular processes involved in secondary injury, it is highly improbable that a single treatment approach will lead to complete restoration of function. The necessity for combination therapies is broadly acknowledged. Therefore, in the present study, we investigate how a combination treatment using adipose AD-MSC conditioned medium and dehydroepiandrosterone affects the locomotor, oxidative stress, and histopathological changes caused by SCI in a rat model.

## 2 Methods and materials

### 2.1 Animals

Adult female Wistar rats (180–200 g) were acquired from the Comparative and Experimental Medical Center at Fasa University of Medical Sciences (FUMS). All animals were maintained under standard circumstances, temperature: 22 ± 2°C; relative humidity: 50 %, which included a temperature of 22 ± 2°C, a relative humidity of 50 %, and a 12-hour light/dark cycle, with unrestricted accessibility to water and food. *In vivo* procedures were performed following ethical standards outlined in the NIH Guide for the Care and Use of Laboratory Animals and ARRIVE Guidelines, with approval from the university's Ethics Committee (ethical code number: IR.BMSU.AEC.1403.026).

### 2.2 Study design

Sixteen female Wistar rats were allocated randomly into five experimental groups (n = 12). Females were chosen due to their consistently low levels of circulating DHEA. In rodents, DHEA is produced in the gonads, specifically by Leydig cells in the testis and Theca cells in the ovary, but not by the adrenals. In females, the low DHEA level produced in the ovary is for local androgen and estrogen production and does not contribute to circulating levels. In contrast, males have higher and more variable circulating DHEA, which serves as a precursor for androgens both in the gonads and in peripheral organs (Nenezic et al., 2023, Imamichi et al., 2016). Rats were divided randomly into the following five groups: 1- LME group, laminectomy was done followed by daily 200 µl vehicle (Dimethyl sulfoxide 1 % (DMSO)) intraperitoneally (IP) for seven days; 2- SCI group, after laminectomy, the compression of spinal cord was done, then the rats received daily 200 µl DMSO 1 % for seven days; 3- SCI-DHEA group, the rats were treated with DHEA daily at a dosage of 30 mg/kg, dissolved in 1 % DMSO, administered IP for seven days after SCI. The DHEA dosage is based on a previous study (Prakash et al., 2019); 4- SCI-CM group, the rats were given AD-MSCs' conditioned medium (200 µl, daily IP) for seven days following SCI. The dosage is based on a previous study (Fontanilla et al., 2015); 5- SCI-DHEA+CM group, the rats received the same mentioned doses of both DHEA and the conditioned medium for seven days after SCI. All groups received their first dose after the SCI surgery. The pro-regenerative and inflammatory responses begin within the first week following SCI, indicating that this timeframe may serve as a valuable opportunity for therapeutic intervention (Devaux et al., 2016). Therefore, we chose to administer treatments to the groups during the first week after SCI.

Locomotor recovery was monitored weekly for 28 days following the induction of SCI. After this period, the rats were sacrificed for the collection of spinal tissue. To accommodate the different tissue processing needed for biochemical and histological assessments, six rats from each group were randomly selected for histological assessment, and the other six were chosen for oxidative stress tests.

### 2.3 Spinal cord injury operation

Thirty minutes before the operation, the antibiotic ceftriaxone (50 mg/kg) was administered to the animals. After anesthetizing the animals with intraperitoneal ketamine (80 mg/kg) and xylazine (10 mg/kg), they were maintained on a thermostatically controlled heating pad, and their backs were disinfected with betadine and alcohol. A 3-cm skin incision was made parallel to the spinous processes of the 11th and 9th thoracic vertebrae. The paravertebral muscles were retracted, and once the spinous process of the 10th thoracic vertebra was visible, it was excised using a micro rongeur. Then, A micromotor (Marathon Escort 2 pro, Saeyang Microtech, Korea) with a dental burr (Meisinger RA #559 Straight Fissure Cross Cut Carbide Bur, Germany) was used to drill the dorsal lamina on both sides. Dorsal lamina drilling was carefully performed to avoid spinal cord injury, using saline to prevent thermal damage. After removing one-third of its thickness, the lamina was fully excised, revealing the intact spinal cord (VS et al., 2019). Spinal cord injury in this study was performed based on an aneurysm clip compression model (Dolan et al., 1980). A mini straight aneurysm clip (Sugita, 100 g force) was placed on the exposed spinal cord and held for 10 s. The creation of a spinal cord lesion was confirmed by observing a hematoma in the spinal cord tissue and observing convulsive movements in the hind limbs and wave-like movements of the tail. After injury, the muscles were sutured using 3–0 Vicryl suture, and the skin was sutured with 4–0 Nylon. After surgery, pain was controlled by administering diclofenac (25 mg/kg). Manual bladder massage of the rats was conducted twice daily until they regained normal urination.

### 2.4 MSC collection from rat adipose tissue

Visceral adipose tissue was surgically excised from the peri-gastric region of the two adult female Wistar rats under aseptic conditions. To purify AD-MSC, adipose tissue was thoroughly rinsed with phosphate buffer containing 2 % antibiotic-antimycotic solution (Gibco). The tissue was enzymatically dissociated using 0.25 % Collagenase type I (Gibco) with gentle agitation for 90 min at 37 °C. After incubation, the digested tissue was filtered through a 100 μm nylon mesh to eliminate remaining tissue fragments. The collected fraction was centrifuged at 1200 rpm for 5 min. The pellet of stromal cells was suspended in a 50/50 mixture of DMEM and Ham’s F-12 (Corning), with the addition of 10 % fetal bovine serum (Gibco) and 1 % penicillin/streptomycin (Gibco), and then transferred to a 25 cm² tissue culture flask. The cells were kept in a humid environment with 5 % CO2 at a temperature of 37 °C. Non-adherent cells were discarded after 2–3 days, and the culture medium was replaced twice a week (Szekiova et al., 2018).

### 2.5 Characterization of AD-MSC phenotype

Flow cytometry was used to characterize adipose mesenchymal stem cells by identifying their lack of hematopoietic stem cell markers, CD34 and CD45, while expressing CD44 and CD90. Antibodies used included PE anti-rat CD90, PE anti-rat CD45, and PE anti-rat CD29 from BioLegend. Samples were analyzed on a FACS Calibur (BD Bioscience) with CellQuest software (Mildmay-White and Khan, 2017).

### 2.6 Gathering conditioned media from AD-MSC

To prepare the conditioned medium, mesenchymal stem cells (10 ^4^ cells/cm²) were cultured in DMEM medium (Corning) without FBS and antibiotics from the third to the fifth passage. Afterward, the cell culture medium was collected and centrifuged at 1500 rpm for 5 min. The supernatant was then centrifuged a second time at 3000 rpm for 3 min, and finally, its supernatant was used as conditioned medium (Yang et al., 2014). All experiments used CM derived from the same batch of AD-MSCs to ensure consistency in the composition of the secretome across all treated animals.

### 2.7 Motor function evaluation

Two tests were conducted following SCI induction to evaluate the motor function of rats: the BBB test and the rotarod test.

The BBB test assesses rat hindlimb movements, paw placement, trunk stability, toe clearance, stepping coordination, and tail position. Blinded observers document weekly scores from 0 (no locomotion) to 21 (normal motor function) (Basso et al., 1995).

In the rotarod test, the rats underwent pre-training on the rotarod device at a speed of 5 rpm for a minimum of 5 min over three consecutive days before the compression injury. The day before SCI induction, the rats were evaluated by gradually increasing the speed from 5 to 20 rpm over a 5-minute period. After 28 days post-SCI, motor performance was evaluated using the same testing conditions (Avila-Martin et al., 2011).

### 2.8 Oxidative stress assessment

On the 28th day after SCI, the animals were euthanized following deep anesthesia. Then, the spinal cord surrounding the injury's epicenter was promptly extracted and rapidly frozen with liquid nitrogen. The frozen spinal was first weighed and then homogenized for approximately 3 min in ice-cold EDTA-potassium phosphate buffer using a Homogenizer (T 10 basic ULTRA-TURRAX, IKA, Germany). Following homogenization, the mixture was centrifuged at 12,000 rpm for 5 min at 4°C. The supernatant was separated and stored at −80°C for colorimetric assays of oxidative stress markers using a microplate reader (BioTek Synergy H1, Agilent, USA).

### 2.9 Malondialdehyde estimation

MDA in the spinal tissue was estimated using the thiobarbituric acid (TBA) reaction method, where the pink color from TBA reacting with MDA is quantified at 532 nm (Ohkawa et al., 1979). TEP (1,1,3,3-Tetraethoxypropane) is used as a standard since it generates MDA upon hydrolysis. MDA equivalents, a marker of lipid peroxidation, were calculated by correlating sample absorbance values with a TMP standard reference curve.

### 2.10 Glutathione estimation

The amount of glutathione (GSH) in the spinal tissue was assessed using the Ellman method (Rahman et al., 2006). In this assay, Ellman’s reagent, which is also referred to as DNTB (5,5′-dithio-bis-[2-nitrobenzoic acid]), interacts with GSH. The resulting complex (5-thionitrobenzoic acid) exhibits a yellow coloration. The absorbance measured at 405 nm is utilized to determine the concentration of GSH in the sample.

### 2.11 Superoxide dismutase and catalase activity

The activities of the enzymes SOD and catalase (CAT) were assessed utilizing Assay Kits (ZellBio GmbH, Germany) following the instructions provided by the manufacturer.

### 2.12 Histopathological examination

#### 2.12.1 Tissue processing method

On the 28th day post-SCI, the animals were sacrificed after being deeply anesthetized. The spinal cord adjacent to the injury (approximately 6 mm) was removed along with the adjacent muscle and skeletal tissues and fixed in 4 % paraformaldehyde for two weeks. Subsequently, the spinal cord was removed and transferred to a 30 % sucrose solution in PBS. After ensuring dehydration, the tissue samples were briefly placed in isopentane solution and then stored at −80°C. In the next step, the tissue was covered with Optimal Cutting Temperature Compound (Tissue-Tek, Sakura, USA). Sections of 40 μm thickness were selected at 200 μm intervals using systematic random sampling with a cryostat (HM525, Thermo Scientific). Finally, sections were mounted on gelatin-coated slides and stained with Luxol Fast Blue and Cresyl Violet.

#### 2.12.2 Stereological quantification

An image sensor was connected to a Nikon Eclipse 200 microscope to acquire images. Live image data was imported into Image J (Fiji) using a webcam capture plugin, then, for quantification, a grid and sampling disector superimposed on the live image (Ghaffari et al., 2024).

**Fig. 1:**
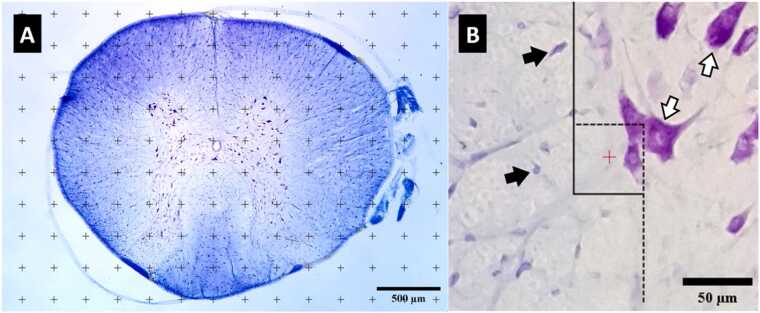
Stereological techniques. **A** represents a 250 × 250 μm grid superimposed on a spinal section, and **B** represents a 50 × 50 μm disector. To ensure unbiased cell counting, the line sides are not counted, and the stroke sides are counted. White arrow: neuron, Black arrow: non-neuron.

The total volume of the spinal cord, along with the spared white and grey matter, as well as the lesion volume, was estimated using the point counting technique based on Cavalieri’s principle. A point-grid system was overlaid onto a live image of each section using the ImageJ grid plugin, and the points within the region of interest were manually counted (Fig. 1A). Then, the volume was estimated from the following equation:

V = (∑P) x (a/p) x T

The total point count is represented by ∑P, while a/p denotes the area linked to each point, and T refers to the section interval. The sampling grid size, set at 250 μm × 250 μm, was fine-tuned to attain a mean Gundersen coefficient of error (CE) of less than 10 % (Gundersen et al., 1999).

To estimate the total number of neuronal cells in the gray matter and non-neuronal cells throughout the entire spinal cord, we first calculated the numerical density of these cells. Next, the total number of cells was calculated by multiplying the density by the reference volume. Cell density was estimated using the optical disector technique. To implement this approach, the live image was calibrated using a Zeiss micrometer under high-power magnification. Subsequently, a precisely unbiased brick (50 μm × 50 μm) was randomly overlaid onto the live image utilizing the Image J disector plugin (Fig. 1B). The microscope was equipped with a microcator (Heidenhain, Germany) for z-direction measurements. Cells were counted along the z-axis using a Nikon Plan Apo 100x/1.40 Oil objective. A guard volume was utilized on either side of the section to prevent artifacts from sectioning. About 200 systematically random locations were sampled from sections of each case to ensure that the coefficient of error remained below 10 % for all groups. The numerical density is determined using this formula:

Nv = ∑Q ∕ (∑P × a(f) × h)

∑Q represents the total number of counted cells, ∑P denotes the total number of counted disectors, a(f) signifies the area of the disector, and h refers to the height of the disector (Howard and Reed, 2004).

**Fig. 2:**
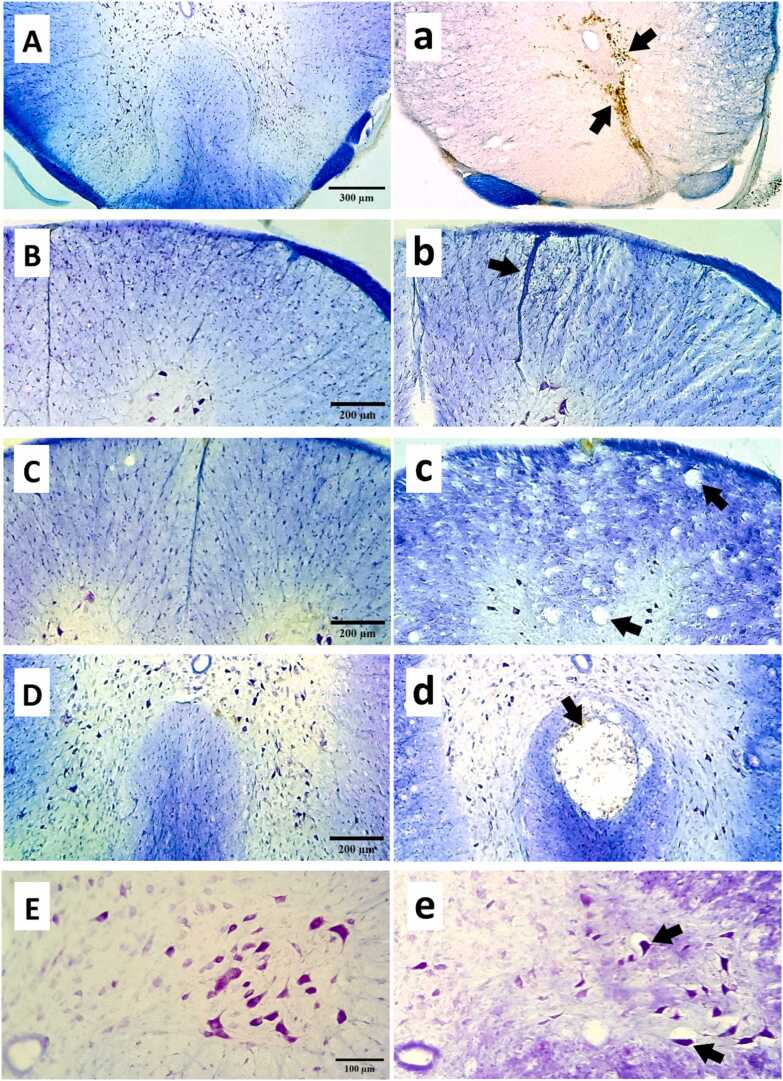
Representative photomicrographs of spinal cord sections stained with Cresyl Violet and Luxol Fast Blue. Uppercase letters represent normal tissue from the laminectomy group, and lowercase letters represent different histopathological states from the SCI group. a: hemosiderin accumulation from the previous hemorrhage; b: vascular congestion, mononuclear infiltration, and edematous tissue; c: vacuolation; d: cyst formation; e: damaged neuron.

Cell types were identified based on variations in size, shape, chromatin arrangement, and Nissl bodies. To identify spinal cells, we employed a protocol focused on their varying staining properties for cell differentiation (Bjugn and Gundersen, 1993). Neuronal nuclei were typically larger than those of glial cells. Glial cells often appeared with unstained cytoplasm. Neurons generally had a prominent nucleolus. Within the dorsal horns, particularly in laminae 1-III, numerous neurons exhibited pronounced indentations in their nuclear membrane. Endothelial cells, along with pericytes and smooth muscle cells, were identified based on their position surrounding a blood vessel or their curved morphology. Cells that were unable to be identified as neurons were classified as non-neuronal cells (Fig. 1b). normal histologic appearance. Characteristics indicative of neuronal damage included eosinophilic cytoplasm, vacuolation, and a pyknotic appearance (loss of nuclear integrity). Cells that demonstrated Nissl substance within the cytoplasm, loose chromatin, and distinct nucleoli were deemed viable (Fig. 2E, e) (Kiziltepe et al., 2004).

#### 2.12.3 Histopathological scoring

Tissue sections were processed with Cresyl Violet and Luxol Fast Blue staining for histopathological observations. Spinal cord injury was graded semiquantitatively by a blinded pathologist using coded slides. Histopathological assessments were graded on a 0–3 scale according to the existence of neuronal injury, vacuolation, cyst formation, hemorrhage, and inflammation (Fig. 2). A score of 0 indicated no evidence, 1 mild, 2 moderate, and 3 severe alterations. These scores were then summed to provide a cumulative score ranging from 0 to 15 (Afshari et al., 2018). For the gray matter, the criteria were as follows: 0, no neuronal injury; 1, 10 % neuronal injury; 2, 10–50 % neuronal injury; and 3, over 50 % neuronal injury. In terms of white matter, the criteria included: 0, no vacuolation; 1, few vacuolations; 2, extensive vacuolation; and 3, widespread vacuolation (Kato et al., 1997). We need to conduct a semiquantitative assessment of neuronal injury, as injured neurons are unevenly distributed; our systematic random sampling method cannot accurately estimate the exact number of injured neurons with a coefficient of error below 10 %. This scoring system is derived from a previous study (Kato et al., 1997). Volume of cavitation, hemosiderin accumulation, and vacuolation were considered in the lesion volume. In the point grid system, the points that collided with the cavities, accumulated hemosiderin, vacuoles, and unstained areas counted for lesion volume estimation.

### 2.13 Statistical evaluation

Data were expressed as mean ± SEM, with a significance level of P < 0.05. Data normality was assessed using the Kolmogorov-Smirnov test. To evaluate changes in the BBB score over the weeks, a two-way repeated-measures analysis of variance (TWRM-ANOVA) was employed, while the one-way ANOVA followed by Tukey’s post hoc test was used for comparing other parameters among different groups. All quantitative analyses were carried out using GraphPad Prism's statistical package (version 8).

## 3 Findings

### 3.1 Characterization of AD-MSC

**Fig. 3:**
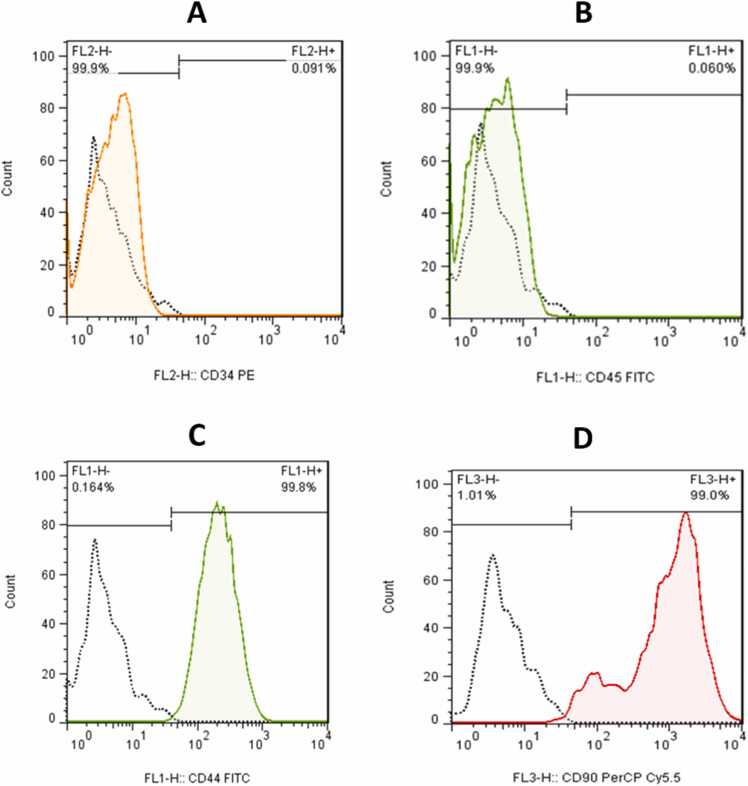
Phenotypic characterization of AD-MSCs by flow cytometry. A: 0.091 % CD34; B: 0.060 % CD45; C: 99.8 % CD44; D: 99 % CD90.

Flow cytometry analysis of cell surface markers of harvested cells at passage 3 revealed the cells had strong expression of MSC markers (CD44 and CD90 >99 %) while having minimal hematopoietic marker expression (CD34 and CD45 <1 %) (Fig. 3).

### 3.2 Basso, Beattie, Bresnahan (BBB) score

**Fig. 4:**
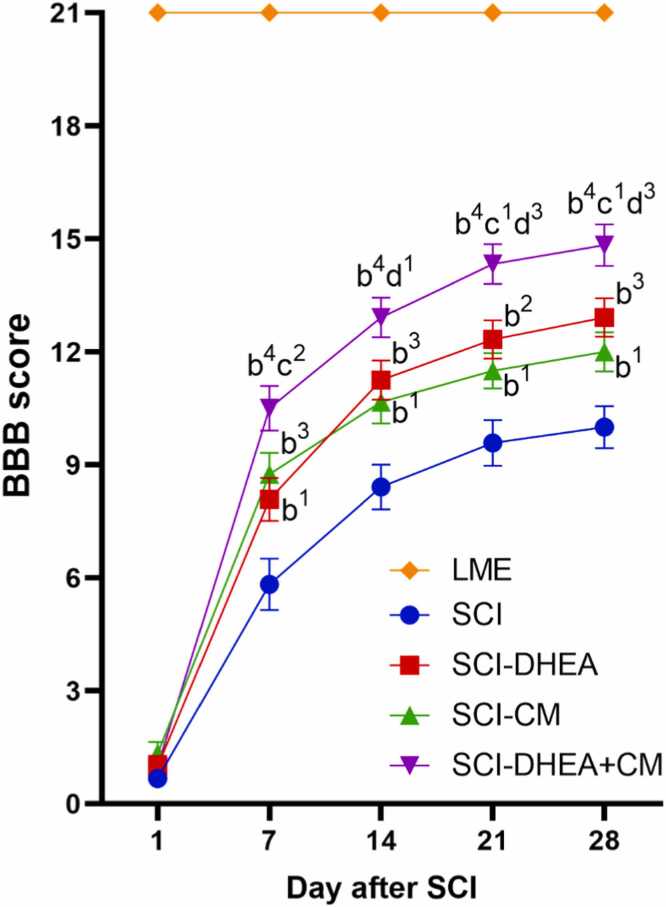
line chart of BBB score of groups in days after SCI. The data were expressed as mean ± SEM, with n = 12, and analyzed using TWRM-ANOVA followed by Tukey’s multiple comparison tests. (b) indicates a comparison with the SCI group, (c) indicates a comparison with the SCI-DHEA group, and (d) indicates a comparison with the SCI-CM group. 4 P < 0.0001; 3 P < 0.001; 2 P < 0.01; and 1 P < 0.05.

As shown in Fig. 4, treated groups have a higher BBB score in comparison with the SCI group from day 7–28 after SCI. On day 7 after SCI, the BBB score of the SCI-DHEA+CM group was significantly higher than the SCI-DHEA group (p < 0.01). On day 14 after SCI, the BBB score of the SCI-DHEA+CM group was significantly higher than the SCI-CM group (p < 0.05). On days 21 and 28 after SCI, the BBB score of the combination treatment group was significantly higher than the single treatment groups (SCI-DHEA, p < 0.05; SCI-CM, p < 0.001).

### 3.3 Fall latency of the rotarod test

**Fig. 5:**
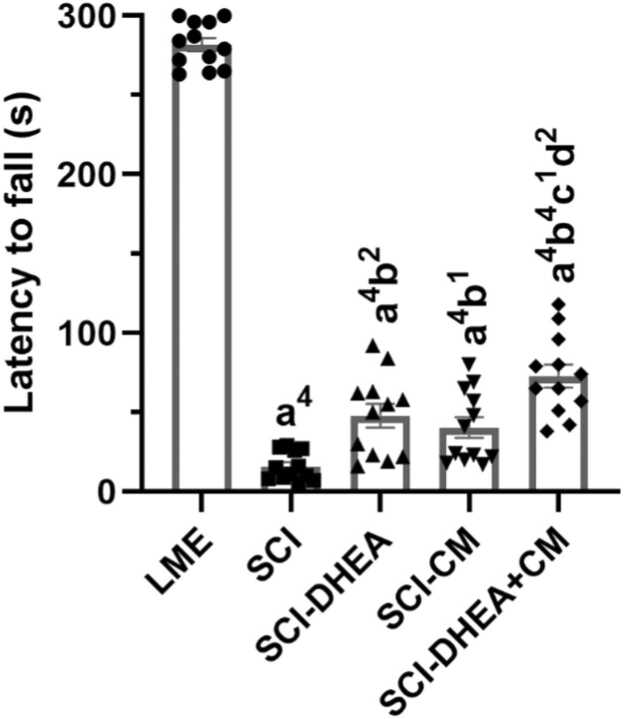
Fall latency of the rotarod test. The data were expressed as mean ± SEM, with n = 12, and analyzed using one-way ANOVA followed by Tukey’s multiple comparison tests. (a) indicates a comparison with the LME group, (b) indicates a comparison with the SCI group, (c) indicates a comparison with the SCI-DHEA group, and (d) indicates a comparison with the SCI-CM group. 4 P < 0.0001; 2 P < 0.01; and 1 P < 0.05.

Analysis of fall latency in the rotarod test indicated that the SCI groups exhibited significantly lower fall latency compared to the LME group (p < 0.0001). Treatment with DHEA (p < 0.01), CM (p < 0.05), and their combination (p < 0.0001) increased fall latency compared to the SCI group (Fig. 5). In comparison between treatment groups, the SCI-DHEA+CM group exhibited a significantly longer fall latency compared to the SCI-DHEA (p < 0.05) and SCI-CM (p < 0.01) groups.

### 3.4 Oxidative stress markers

#### 3.4.1 Glutathione concentration

**Fig. 6:**
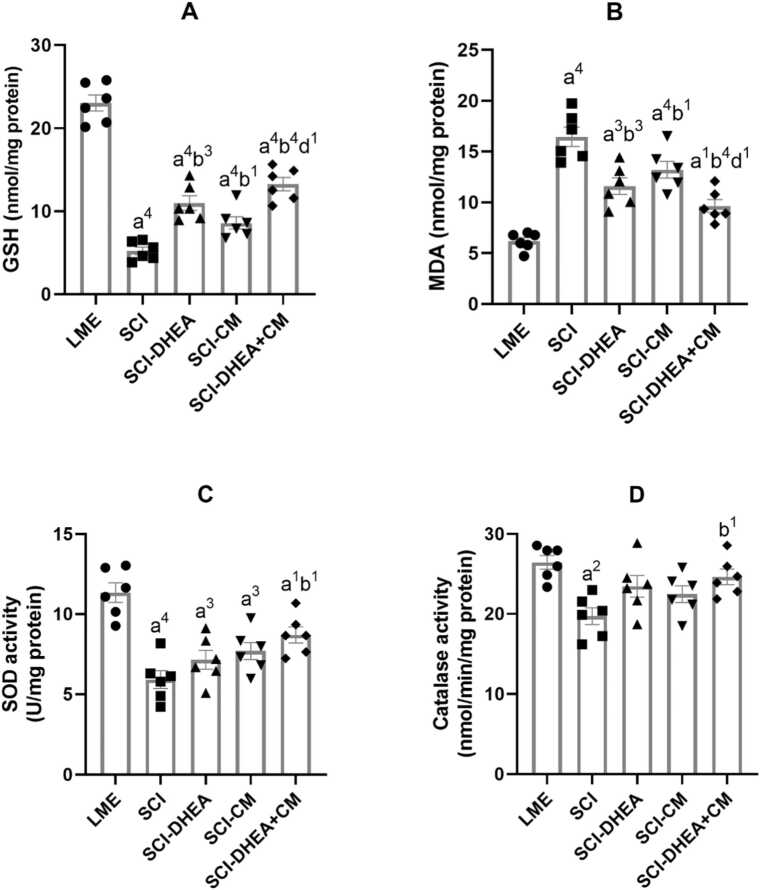
Oxidative stress markers of the spinal. **A:** GSH concentration; **B:** MDA concentration; **C:** SOD activity; **D:** catalase activity. The data were expressed as mean ± SEM, with n = 6, and analyzed using one-way ANOVA followed by Tukey’s multiple comparison tests. (a) indicates a comparison with the LME group, (b) indicates a comparison with the SCI group, and (d) indicates a comparison with the SCI-CM group. 4 P < 0.0001; 3 P < 0.001; 2 P < 0.01; and 1 P < 0.05.

The concentration of spinal GSH in the SCI groups was significantly lower than in the LME group (p < 0.0001). In comparison to the SCI group, treatment with DHEA (p < 0.001), CM (p < 0.05), and their combination (p < 0.0001) significantly increased GSH levels in the spinal cord (Fig. 6A). Moreover, the SCI-CM+DHEA group had higher GSH than the SCI-CM group (p < 0.05).

#### 3.4.2 Malondialdehyde concentration

As shown in Fig. 6B, the concentration of MDA in the spinal cord of the SCI groups was significantly higher than in the LME group. Treatment with DHEA (p < 0.001), CM (p < 0.05), and their combination (p < 0.0001) significantly reduced MDA concentration in the spinal cord (Fig. 6B). In comparison between treatment groups, the SCI-CM+DHEA group had lower MDA than the SCI-CM group (p < 0.05).

#### 3.4.3 Superoxide dismutase activity

The SCI groups displayed significantly lower spinal cord SOD activity compared to the LME group. Treatment with DHEA and CM significantly increased SOD activity of the spinal compared to the SCI group (p < 0.05; Fig. 6C).

#### 3.4.4 Catalase activity

The spinal cord of the SCI group had significantly lower catalase activity in comparison to the LME group (p < 0.01). The combination treatment prevented the reduction and showed higher catalase activity compared to the SCI group (p < 0.05; Fig. 6D).

### 3.5 Histopathological results

#### 3.5.1 Spinal volume

**Fig. 7:**
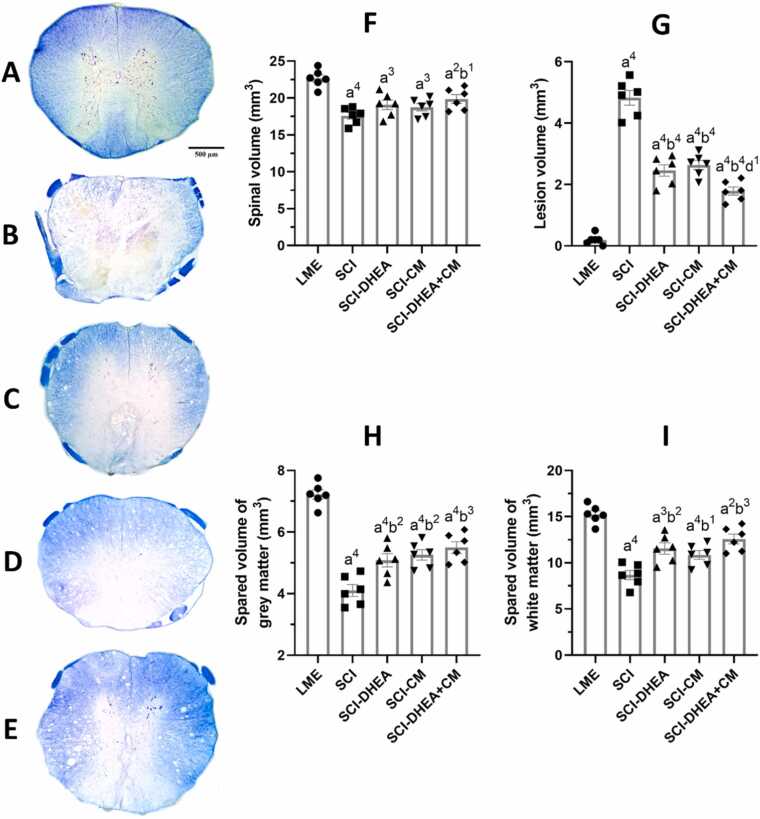
Estimated volumes with stereology. A-E: Represent spinal sections from close to the injury's epicenter in the LME, SCI, SCI-DHEA, SCI-CM, and SCI-DHEA+CM groups, respectively. F: Spinal volume, G: Lesion volume, H: Spared volume of grey matter, I: Spared volume of white matter. The data were expressed as mean ± SEM, with n = 6, and analyzed using one-way ANOVA followed by Tukey’s multiple comparison tests. (a) reveals a comparison with the LME group, (b) reveals a comparison with the SCI group, and (d) reveals a comparison with the SCI-CM group. 4 P < 0.0001; 3 P < 0.001; 2 P < 0.01; and 1 P < 0.05.

As shown in Fig. 7F, SCI induction significantly mitigated the total volume of the spinal cord compared to the LME group. The SCI-DHEA+CM group exhibited a significantly higher spinal cord volume compared to the SCI group (p < 0.05).

#### 3.5.2 Lesion volume

In comparison to the LME group, the SCI groups have significantly higher lesion volume (p < 0.0001). Treatment with DHEA, CM, and their combination decreased lesion volume compared to the SCI group (p < 0.0001). In comparison between treatment groups, the SCI-DHEA+CM group had lower lesion volume than the SCI-CM group (p < 0.05; Fig. 7G).

#### 3.5.3 Spared volume of grey matter

The volume of spared spinal gray matter in the SCI groups was significantly lower compared to the LME group (p < 0.0001). Treatment with DHEA, CM, and their combination resulted in increased grey matter volume compared to the SCI group (p < 0.01; Fig. 7H).

#### 3.5.4 Spared volume of white matter

SCI groups had significantly lower white matter volume compared to the LME group. Treatment with DHEA (p < 0.01), CM (p < 0.05), and DHEA+CM (p < 0.001) increased white matter volume compared to the SCI group (Fig. 7I).

#### 3.5.5 Total number of neurons in the spared grey matter

**Fig. 8:**
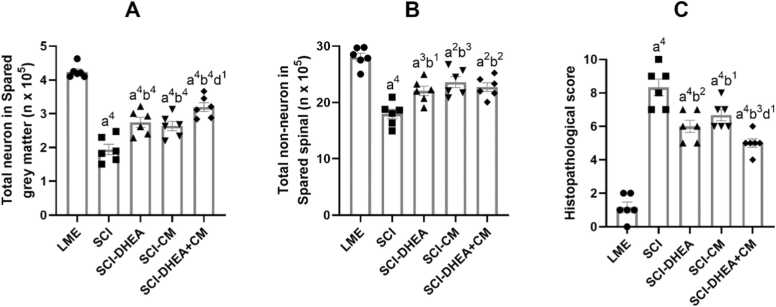
Quantitative histological results. A: total number of neurons in spared grey matter, B: Total number of non-neuronal cells in spared spinal, C: Sum of histopathological scores. The data were expressed as mean ± SEM, with n = 6, and analyzed using one-way ANOVA followed by Tukey’s multiple comparison tests. (a) indicates a comparison with the LME group, (b) indicates a comparison with the SCI group, and (d) indicates a comparison with the SCI-CM group. 4 P < 0.0001; 3 P < 0.001; 2 P < 0.01; and 1 P < 0.05.

As shown in Fig. 8A, SCI induction significantly decreased the total number of neurons in the spared grey matter compared to the LME group (p < 0.0001). Treatment groups had higher neuron counts in spared grey matter than the SCI group (p < 0.0001). In comparison between treatment groups, the SCI-DHEA+CM group had higher neuron counts in spared grey matter than the SCI-CM group (p < 0.05).

#### 3.5.6 Total number of non-neuronal cells in the spared spinal

There is a significant reduction in the total number of spared spinal non-neuronal cells in the SCI groups compared to the LME group. In comparison with the SCI group, treatment with DHEA (p < 0.05), CM (P < 0.001), and DHEA+CM (p < 0.01) significantly increased the total number of non-neuronal cells in the spared spinal (Fig. 8B).

#### 3.5.7 Histopathological score

The sum of pathological scores indicated that the SCI groups had significantly higher scores than the LME group (p < 0.0001). Treatment groups had lower scores than the SCI group (SCI-DHEA, p < 0.01; SCI-CM, p < 0.05; SCI-DHEA+CM, p < 0.001). The SCI-DHEA+CM group scored significantly lower than the SCI-CM group (p < 0.05; Fig. 8 C).

## 4 Discussion and conclusion

AD-MSCs generate a wide variety of chemokines, cytokines (both pro- and anti-inflammatory cytokines), antioxidative agents, adipokines, pro-angiogenic agents, anti-apoptotic substances, growth factors (including IGF-1, VEGF, HGF, EGF, FGF 21, and FGF-2), neurotrophic factors (such as NGF and BDNF), and interleukins (like IL-6, IL-7, IL-8, and IL-11) (Bunnell, 2021, Mirzaei et al., 2025). They also possess various types of extracellular vesicles along with miRNA that exhibit neuroprotective and neurotrophic properties (Fang et al., 2019, Baez-Jurado et al., 2019). This has sparked interest in MSC-derived acellular products (such as secretome, various types of extracellular vesicles, and conditioned culture medium) as potential therapeutic options for lesions and disorders of the nervous system.

The present study scrutinized the impact of ADMSC-CM and DHEA on locomotor, oxidative stress, and histopathological changes induced by SCI in rats. We demonstrate for the first time that treatment with ADMSC-CM can facilitate locomotor recovery, decrease oxidative stress, reduce lesion volume, prevent the loss of neuronal and non-neuronal cells, and improve the histopathological defects induced by SCI. Moreover, we discovered that combining ADMSC-CM with DHEA produced stronger effects than using ADMSC-CM alone.

Research indicated that DHEA therapy improved recovery from locomotor disabilities caused by SCI and expanded the region of white matter preserved at the site of injury while diminishing the extent of reactive gliosis around the lesion (Fiore et al., 2004). Moreover, several studies demonstrated that DHEA could prevent neuronal damage through oxidative stress regulation in various CNS injury models (Prakash et al., 2019, Aragno et al., 2000, Chakraborti et al., 2011). In agreement with these studies, we found that DHEA treatment improved locomotor recovery after SCI, as evaluated by the BBB (Fig. 4) and rotarod (Fig. 5) tests. Also, DHEA prevents oxidative stress (Fig. 6A, B), reduces lesion volume (Fig. 7G), preserves white and grey matter (Fig. 7H, I), protects neuron and non-neuron loss (Fig. 8 A, B), and decreases histopathological score (Fig. 8 C) of the spinal cord tissue.

DHEA is a neurosteroid known to act as a neurotropic or neuroprotective agent that protects the CNS from various types of damage, including excitotoxicity (Wojtal et al., 2006). It was shown that DHEA alleviates oxidative stress by triggering the Nrf2 signaling pathway (Jeon et al., 2015). Scientific investigations have suggested that DHEA treatment may potentiate NO synthesis through elevated stabilization and expression of endothelial NO synthase (eNOS). Moreover, DHEA triggers eNOS by a non-transcriptional mechanism that is related to MAPK and ERK1/2 signaling (Simoncini et al., 2003). NO regulates CNS blood flow, and some of the neurotransmitters, like GABA (Vincent and Kimura, 1992, Trabace and Kendrick, 2000). Some reported effects may have been caused by other circulating steroids, as circulating DHEA may be converted in peripheral organs into a variety of different steroid hormones, such as androgens and estrogens. Anti-inflammatory effects of DHEA have also been found in cellular and animal models of the CNS (Nenezic et al., 2023, Loria et al., 1996). Therefore, DHEA may play its protective role through eNOS activation, oxidative stress regulation, and anti-inflammatory effects.

Inflammation is a key regulator of the SCI secondary damage cascade, which can be both beneficial and detrimental. Initially, inflammatory signals chemotax neutrophils, microglia, and circulating macrophages to the injury site. Monocyte-derived cells remove toxic cellular debris and produce substances that may protect specific parts of the damaged microenvironment. However, these cells create large levels of inflammatory cytokines, which can be harmful to adjacent cells and hinder regeneration (Rust and Kaiser, 2017, Azimi et al., 2025, Khalaji et al., 2024, Anaraki et al., 2024). It was shown that conditioned medium from neural stem cells decreased the expression of inflammatory cytokines in both activated macrophages and tissue of the injured spinal cord while preserving macrophage beneficial phagocytic ability. Through this mechanism, it improved motor function and regeneration of injured spinal cord tissue (Cheng et al., 2017)

A study demonstrated that treatment with conditioned Medium derived from BM-MSCs facilitates motor recovery after SCI and induces pro-angiogenic and tissue-protective effects (Cantinieaux et al., 2013). Another study showed that intrathecal treatment with mesenchymal Stromal Cells Conditioned Medium enhanced locomotor recovery, increased spared spinal cord tissue, and decreased inflammation (Cizkova et al., 2018). In line with these studies, we found that ADMSC-CM improved functional recovery (Figs. 4, 5), reduced lesion volume (Fig. 7G), cell loss, and pathological defects (Fig. 8). We could not find any studies that investigate the effects of treatment with conditioned medium derived from AD-MSC in the SCI model; however, a study demonstrated that AD-MSC transplantation after SCI improved motor recovery through axon preservation and vascularization enhancement (Takahashi et al., 2018). Moreover, an *in vitro* study showed that ADMSC-CM increased the survival of neurons exposed to medium derived from the injured spinal cord (Szekiova et al., 2018). Another study indicated that ADMSC-CM treatment increased the number of spinal neurons and reduced the activation of microglia and astrocytes in an amyotrophic lateral sclerosis transgenic mouse model (Fontanilla et al., 2015).

The spinal cord is especially susceptible to oxidative stress because of elevated levels of polyunsaturated fatty acids, reactive oxygen metabolites, metabolic activity, and relatively low antioxidant capacity. Excessive oxidative stress is a key feature of the secondary phase of SCI. Numerous studies have shown that the production of reactive oxygen species (ROS) and subsequently oxidative stress are typical occurrences in SCI (Jia et al., 2012). We found that treatment with ADMSC-CM after SCI decreased the oxidative stress of the spinal cord (Fig. 6). Several studies demonstrated that treatment with conditioned medium derived from MSCs decreased oxidative stress in various CNS injury models (Jiang et al., 2019, Huang et al., 2021, Zangiabadi et al., 2024).

Because various molecular processes are involved in secondary injury, a single treatment approach may not be enough for the restoration of function; therefore, in the current work, we investigate the combination treatment of ADMSC-CM and DHEA. We found combination treatment had stronger effects on locomotor recovery in comparison with ADMSC-CM alone (Figs. 4, 5). Also, it had a significantly better histological profile than the ADMSC-CM-treated group (Fig. 7, 8). These stronger influences may stem from targeting various signaling pathways. For example, ADMSC-CM contains some proinflammatory cytokines, which may worsen the microenvironment of the injured spinal cord. DHEA has anti-inflammatory effects and could prevent this effect. Moreover, DHEA, through the Nrf2 signaling pathway, increased the antioxidative capacity of the spinal cord. In agreement, we found that, compared to single therapy, the combined treatment of ADMSC-CM with DHEA significantly reduced oxidative stress (Fig. 6A, B) and increased antioxidant enzyme activity (Fig. 6C, D). Therefore, the better functional and histological profile of the combination treatment may be related to its stronger effect on oxidative stress reduction.

## Author contributions

F.M and G.K contributed to the acquisition, analysis, and interpretation of data for the work. F.M and M.R contributed to the write-up of the draft of article. G.K and F.R.T: Contributed to the supervision, editing and designed the framework of the manuscript. All authors read and approved the final version of the manuscript.

## CRediT authorship contribution statement

**Farrokh Modarresi:** Writing – original draft, Methodology, Data curation, Conceptualization. **Fatemeh Rezaei-Tazangi:** Writing – review & editing, Supervision, Project administration. **Gholam Reza Kaka:** Writing – original draft, Methodology, Data curation. **Mehdi Raei:** Writing – original draft, Methodology, Investigation.

## Informed consent

Not applicable.

## Ethics approval

This study was approved by Ethic committee of Baqiyatallah University of Medical Sciences, Tehran, Iran.

## Funding

This work financially supported by Baqiyatallah University of Medical Sciences, Tehran, Iran.

## Conflicts of Interest

The authors declare no competing interests.

## Data Availability

The datasets used and/or analyzed during the current study are available from the corresponding author upon reasonable request.
